# How immune dynamics shape multi-season epidemics: a continuous-discrete model in one dimensional antigenic space

**DOI:** 10.1007/s00285-024-02076-x

**Published:** 2024-03-27

**Authors:** M. G. Roberts, R. I. Hickson, J. M. McCaw

**Affiliations:** 1 New Zealand Institute for Advanced Study and the Infectious Disease Research Centre, Massey University, Auckland, New Zealand; 2 Health and Biosecurity, CSIRO, Townsville, QLD 4814 Australia; 3 Australian Institute of Tropical Medicine and Hygiene, and College of Public Health, Medical and Veterinary Sciences, James Cook University, Townsville, QLD 4814 Australia; 4 School of Mathematics and Statistics, Faculty of Science, University of Melbourne, Melbourne, VIC 3010 Australia; 5 Centre for Epidemiology and Biostatistics, Melbourne School of Population and Global Health, Faculty of Medicine, Dentistry and Health Sciences, University of Melbourne, Melbourne, VIC 3010 Australia

**Keywords:** Epidemiological modelling, Discrete dynamics, Dynamical systems, Seasonal influenza, SARS-CoV-2, 37N25, 92B

## Abstract

We extend a previously published model for the dynamics of a single strain of an influenza-like infection. The model incorporates a waning acquired immunity to infection and punctuated antigenic drift of the virus, employing a set of coupled integral equations within a season and a discrete map between seasons. The long term behaviour of the model is demonstrated by examples where immunity to infection depends on the time since a host was last infected, and where immunity depends on the number of times that a host has been infected. The first scenario leads to complicated dynamics in some regions of parameter space, and to regions of parameter space with more than one attractor. The second scenario leads to a stable fixed point, corresponding to an identical epidemic each season. We also examine the model with both paradigms in combination, almost always but not exclusively observing a stable fixed point or periodic solution. Adding stochastic perturbations to the between season map fails to destroy the model’s qualitative dynamics. Our results suggest that if the level of host immunity depends on the elapsed time since the last infection then the epidemiological dynamics may be unpredictable.

## Introduction

Respiratory viral illnesses typically cause regular seasonal epidemics in temperate climates. The influenza viruses in particular display strong seasonal behaviour (Webster et al. 2013), but other viruses such as RSV, coronaviruses and rhinoviruses are also etiological causes of seasonal acute respiratory illness (Howard et al. 2013; Mandell 2005). As SARS-CoV-2, the virus that causes Covid-19, transitions out of the pandemic phase—characterised by transient dynamics exhibiting large scale oscillations driven by a range of biological, behavioural and environmental factors—it too may settle into a seasonal cyclic pattern.

Viral infection stimulates the human immune system. Taking influenza as an example, infection elicits a strain-specific antibody immune response, that contributes to the resolution of infection and provides long lasting strain-specific protection such that subsequent exposure is unlikely to result in a productive infection. A similar immune response occurs for other pathogens, including SARS-CoV-2 (Dan et al. 2021; Guo et al. 2022), albeit with evidence that strain-specific immunity may also wane through time (Khoury et al. 2021). Strong strain-specific immunity, combined with the inherent erroneous replication of RNA viruses (Duffy 2018) drives viral evolution (entitled ‘antigenic drift’ for influenza), selecting for immune-escape variants (Guarnaccia et al. 2013). In consequence, for many viral infections, while strain-specific immunity is long (perhaps even life-long), protection against the circulating viral strains may be short lived (Fonville et al. 2014; Grenfell et al. 2004; Smith et al. 2004). We are thus infected with influenza or the common cold on multiple occasions during our life. SARS-CoV-2 shows clear signs of also establishing such transmission patterns (Callaway 2022), which are unsurprising on theoretical grounds (Lavine et al. 2021).

From a dynamical systems perspective, multi-season epidemic dynamics arise from the gain of immunity as a particular strain spreads, and the loss of protection due to the induced selection pressure on the virus leading to the emergence of new immune variants, and/or waning of the host’s strain specific immune response. Efforts to understand this characteristic gain and loss of immunity, and the characteristic dynamics of these systems have been the focus of a number of mathematical models. Kucharski et al. (2015, 2018) investigated antibody dynamics, and how previous exposure influences a host’s response to a new strain, but did not consider the consequential epidemic dynamics. Andreasen (2003) modelled multi-season epidemic dynamics, and in particular a scenario in which variable infectiousness was determined by the time since exposure. In earlier work, through construction of a hybrid continuous-time–discrete-map model, we have considered how immunity obtained in a previous season, and possibly lost in the inter-epidemic period, influences multi-season epidemic dynamics (Roberts et al. 2019). For this model the within season epidemic was represented by a system of ordinary differential equations and the between season changes by a discrete map. Hence a fixed point of the map was equivalent to a periodic solution of the model. Our analysis demonstrated that epidemic dynamics were highly sensitive to parameter choices, with steady-state (i.e. period 1), periodic and complicated dynamics displayed. In another study Kucharski et al. (2016) introduced a general framework for multi-strain epidemic dynamics, in which cross-immunity between strains was modelled based on past exposure history. Noting the combinatorial complexity of considering all possible exposure histories (a challenge that is playing out with SARS-CoV-2 right now (Vattiatio et al. 2022)), they proposed two alternative approaches to reducing the dimensionality of the general system, known as ‘exposure’ and ‘history’ based models (Kucharski et al. 2016).

In the present paper we provide a general model for the within- and between- season dynamics of a respiratory virus. We maintain the structure used by Andreasen (2003) and in our previous work (Roberts et al. 2019): the within season dynamics are represented by a continuous model on $t\in \left( 0,\infty \right)$, and the between season dynamics by a discrete map. First we present a within-season Kermack-McKendrick type model, with the host’s susceptibility to infection taking values from a continuous density. We present a stochastic model for the between-season dynamics, reflecting population turnover and loss of immunity due to waning protection and antigenic drift. We then specialise the model, with the host susceptibility taking discrete values and deterministic between-season dynamics. In the same vein as Kucharski et al. (2016), the long term behaviour of this model is demonstrated by examples where immunity to infection depends on the time since the host was last infected, and where immunity depends on the number of times that a host has been infected.

## The within season model

We assume a population of fixed size. Let the proportion of hosts who have never been infected with the virus at time *t* be $S^\emptyset (t)$. We assume that those that have been infected have a lesser susceptibility to infection with the virus. We index that susceptibility by a variable $\theta$, so that when experiencing the same force of infection, the probability that an individual becomes infected is $k(\theta )$ times the probability that an individual in $S^\emptyset$ would be infected if exposed to the same degree. We assume $k(0)\leqslant 1$ and $k^{\prime }(\theta )\leqslant 0$, where the prime denotes the derivative. We restrict $\theta$ to the range $\left[ 0,1\right]$, so that the proportion of the population with $\theta$ in the range $\left[ a,b\right]$ at time *t* is $\int _a^b S(t, \theta )\,\text{ d }\theta$ and $S^\emptyset (0)+\int _0^1\,S(0, \theta )\,\text{ d }\theta =1$. The model does not have a removed compartment as such, although we can model removal by setting $k(\theta )=0$ in some interval $\theta \in \left( 1-\epsilon ,1\right]$ for $0<\epsilon \ll 1$.

Let an epidemic take place within a time period represented by $t\in \left[ 0,\infty \right)$, in the sense that the first transmission of infection occurs at or after $t=0$, and all transmission has ceased as $t\rightarrow \infty$. During an epidemic the proportion of hosts who are fully susceptible and the density of hosts with susceptibility index $\theta$ decrease according to

$$\begin{aligned} S^\emptyset (t)= S^\emptyset (0)-\int _0^t \imath _\emptyset (t^{\prime })\,\text{ d }t^{\prime } \qquad S(t,\theta ) = S(0,\theta )-\int _0^t \imath (t^{\prime },\theta )\,\text{ d }t^{\prime } \end{aligned}$$

where $\imath _\emptyset (t)$ is the incidence of infection in fully susceptible hosts, and $\imath (t,\theta )$ is the incidence of infection in hosts with susceptibility $\theta$ at time *t*. If the epidemic is precipitated by imported cases with incidence $\jmath _\emptyset (t)$ or $\jmath (t,\theta )$, according to their prior status, and the probability mass function of infectiousness (PMF) with time since infection is $p(\tau )$, then

1 $$\begin{aligned} \imath _\emptyset (t)&= \jmath _\emptyset (t) +\mathcal {R}_0S^\emptyset (t)\Lambda (t) \nonumber \\ \imath (t,\theta )&=\jmath (t,\theta )+ \mathcal {R}_0k(\theta )S(t,\theta ) \Lambda (t) \end{aligned}$$

where $\mathcal {R}_0$ is the basic reproduction number (Diekmann et al. 2013) and the force of infection is

2 $$\begin{aligned} \Lambda (t)&= \int _0^t p(\tau )\left( \imath _\emptyset (t-\tau )+\int _0^1\imath (t-\tau ,\theta )\,\text{ d }\theta \right) \,\text{ d }\tau \nonumber \\&= \int _0^t p(t-\tau )\left( \jmath _\emptyset (\tau )+\int _0^1\jmath (\tau ,\theta )\,\text{ d }\theta \right) \,\text{ d }\tau \nonumber \\&\qquad +\mathcal {R}_0\int _0^t p(t-\tau )\left( S^\emptyset (\tau )+\int _0^1k(\theta )S(\tau ,\theta )\,\text{ d }\theta \right) \Lambda (\tau ) \,\text{ d }\tau \end{aligned}$$

For small *t* we approximate

$$\begin{aligned} \Lambda (t)&= \int _0^t p(t-\tau )\left( \jmath _\emptyset (\tau )+\int _0^1\jmath (\tau ,\theta )\,\text{ d }\theta \right) \,\text{ d }\tau \\&\quad +\mathcal {R}_0\left( S^\emptyset (0)+\int _0^1k(\theta )S(0,\theta )\,\text{ d }\theta \right) \int _0^t p(t-\tau )\Lambda (\tau ) \,\text{ d }\tau \end{aligned}$$

As $\int _0^\infty p(t)\,\text{ d }t=1$, the epidemic *takes off* if

$$\begin{aligned} \mathcal {R}=\mathcal {R}_0\left( S^\emptyset (0)+\int _0^1k(\theta )S(0,\theta )\,\text{ d }\theta \right) >1 \end{aligned}$$

The susceptibility profile of the population at the end of an epidemic may be found from $S^\emptyset (\infty )=S^\emptyset (0) e^{-\mathcal {R}_0\mathcal {P}}$ and $S(\infty ,\theta )=S(0,\theta ) e^{-\mathcal {R}_0k(\theta )\mathcal {P}}$, where $\mathcal {P}$ is the final size of the epidemic (proportion of the population infected throughout the epidemic) and $\mathcal {P}$ solves

3 $$\begin{aligned} \mathcal {P}=S^\emptyset (0)\left( 1-e^{-\mathcal {R}_0\mathcal {P}}\right) +\int _0^1S(0,\theta )\left( 1-e^{-\mathcal {R}_0k(\theta )\mathcal {P}}\right) \,\text{ d }\theta \end{aligned}$$

The derivation of Eq. 3 may be found in Appendix A.

## The stochastic between season model

Let the PMF of $\theta$ post-infection for those that were infected in a season and had previously not been infected be $f(\theta )$. Let the PMF of $\theta$ post-infection for those that were infected in a season and had previous level of immunity $\xi$ be $g(\theta ,\xi )$. Finally, let the PMF at the end of the season for those that were not infected and had previous level of immunity $\xi$ be $h(\theta ,\xi )$. By definition $\int _0^1 f(\theta )\text{ d }\theta =1$, $\int _0^1\,g(\theta ,\xi )\text{ d }\theta =1$, and $\int _0^1 h(\theta ,\xi )\text{ d }\theta =1$. As all those who are infected within a season have increased (strictly not decreased) their level of immunity to infection, we have $f(0)=0$ and $g(\theta ,\xi )=0$ if $\theta <\xi$. Those who are not infected within a season do not increase their level of immunity to infection, hence $h(\theta ,\xi )=0$ if $\theta >\xi$. In the absence of population turnover (i.e. demographic processes of births and deaths) and antigenic drift the PMF of susceptibility at the beginning of the next season would be $F(\theta )+G(\theta )+H(\theta )$ where

$$\begin{aligned} F(\theta )&= f(\theta ) \left( S^{\emptyset }(0)-S^{\emptyset }(\infty )\right) \\ G(\theta )&= \int _0^\theta g(\theta ,\xi )\left( S(0,\xi )-S(\infty ,\xi )\right) \text{ d }\xi \\ H(\theta )&= \int _\theta ^1 h(\theta ,\xi )S(\infty ,\xi )\text{ d }\xi \end{aligned}$$

Now use the subscript *n* to signify this season (prior to the epidemic if it occurs), and $n+1$ for next season. Allow a proportion $1-c$ of the population to be replaced with fully susceptible hosts between seasons, to allow for population turnover and antigenic drift. The initial conditions for next season are

$$\begin{aligned} S_{n+1}(0,\theta )&=c\left( F_n(\theta )+G_n(\theta )+H_n(\theta )\right) \\ S^\emptyset _{n+1}(0)&=1-\int _{0+}^1 S_{n+1}(0,\theta ) \,\text{ d }\theta \end{aligned}$$

The lower limit of the integral above reflects that any individual for whom $\theta =0$ is assumed to have the same susceptibility as those in $S^\emptyset$, and the conservation rule allocates them to that compartment. That situation can only arise in the model through the action of the integral with kernel $h(\theta ,\xi )$.

The process outlined above determines the profile of population susceptibility at the start of year $n+1$. The relative susceptibility function $k(\theta )$ then determines the dynamics during that year. The conditions on *k* are $k(0)\leqslant 1$, $k^{\prime }(\theta )\leqslant 0$ for $\theta \in \left( 0,1\right)$ and $k(1)\geqslant 0$. Suitable example functions include $k(\theta )=A\left( 1-\theta ^r\right)$ for $r>0$ and $A\leqslant 1$; $k(\theta )=A\cos (\theta ^r\pi /2)$; and $k(\theta )=A\left( e^{{-r\theta }}-e^{-r}\right) /\left( 1-e^{-r}\right)$, all of which have $k(1)=0$.

Some examples of the functions *f*, *g* and *h* are discussed below. We first suggest suitable functions that could apply if the time since the most recent infection determines a host’s susceptibility. We then suggest functions that could apply if the number of past infections determines a host’s susceptibility. Finally, we discuss the general case when both of these mechanisms apply.

### The time since most recent infection determines susceptibility

For this example all those who are infected during the year increase their level of immunity to $\theta =1$, the maximum level, and those not infected reduce their level of immunity. An example function for *f* could be

$$\begin{aligned} f(\theta )= \left\{ \begin{array}{rcl} \dfrac{\left( \theta -1+\epsilon \right) ^{a}\left( 1-\theta \right) ^{b}}{\epsilon ^{a+b+1}B(a+1,b+1)}: &{} &{} 1-\epsilon<\theta <1 \\ 0: &{} &{} \text{ otherwise } \end{array} \right. \end{aligned}$$

where $0<\epsilon \ll 1$, *a* and *b* are positive constants, and *B* is a beta function

$$\begin{aligned} B(a+1,b+1)=\int _0^1 \theta ^{a}\left( 1-\theta \right) ^{b}\,\text{ d }\theta \end{aligned}$$

We have $\lim _{\epsilon \rightarrow 0}f(\theta )=\delta (1-)$, a Dirac delta function. In addition, $g(\theta ,\xi )=f(\theta )$ with the added constraint that $\epsilon <1-\xi$ to ensure that $\theta >\xi$. An example function for *h* could be

4 $$\begin{aligned} h(\theta ,\xi )= \left\{ \begin{array}{rcl} \dfrac{\left( \theta -\left( y-\epsilon \right) \xi \right) ^{a}\left( \left( y+\epsilon \right) \xi -\theta \right) ^{b}}{\left( 2\epsilon \xi \right) ^{a+b+1}B(a+1,b+1)}: &{} &{} \left( y-\epsilon \right) \xi<\theta <\left( y+\epsilon \right) \xi \\ 0: &{} &{} \text{ otherwise } \end{array} \right. \end{aligned}$$

This specifies a function in the $\left( \theta ,h\right)$ plane centred at $\theta =y\xi$ with base $2\epsilon \xi$ that integrates to one. The positive constants *a* and *b* do not necessarily take the same values as in the definitions of $f(\theta )$ and $g(\theta ,\xi )$ above. In general, *y* could be a function of $\xi$. To ensure that $\theta >0$ we require $y>\epsilon$, and to ensure that $\theta$ is reduced in the absence of infection we require $y+\epsilon <1$. In the limit $\epsilon \rightarrow 0$ we obtain $h(\theta ,\xi )=\delta (\theta -y\xi )$.

**Fig. 1:**
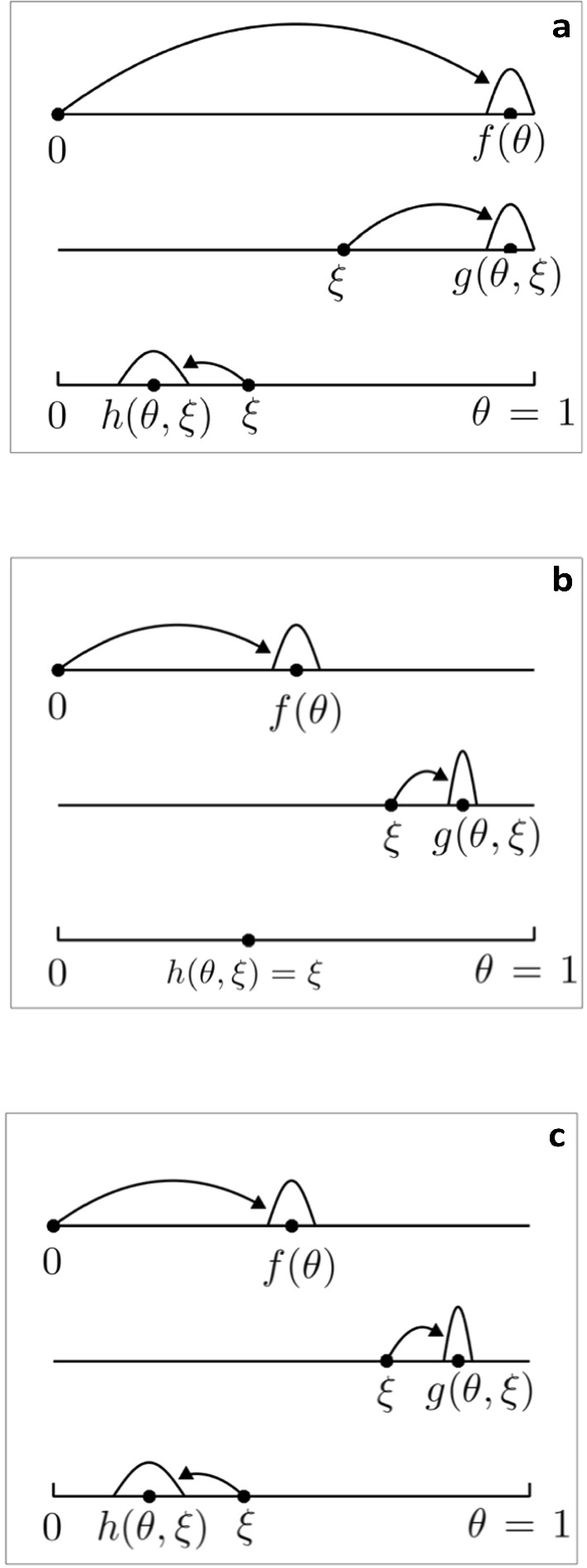
Illustration of the modes of action used to model the immune response. **a** the time since last infection determines immunity; **b** the number of infections determines immunity; **c** the general case where the two mechanisms combine. The function $f(\theta )$ models the response of previously uninfected hosts to infection, the function $g(\theta ,\xi )$ models the response of previously infected hosts to infection, and the function $h(\theta ,\xi )$ models the loss of immunity in the absence of infection

The actions of *f*, *g* and *h* are illustrated in Fig. 1a. We have not discussed how the value of $\theta$ may be determined by the PMFs *f*, *g* and *h*. For a purely deterministic model we would take the limits $\epsilon \rightarrow 0$, realising delta functions. In this situation, and where *y* is a constant, the only levels of $\theta$ realised are $\theta =1$ and $\theta =y^n$, where the last infection occurred $n+1$ seasons ago. For this limiting case, if $\theta$ does not vary continuously at some initial time, it can only take discrete values at future times.

### The number of infections determines susceptibility

For this example an individual’s immunity level is increased at each infection until a maximum value $\theta =1$ is reached. Those who were infected during the year increase their level of immunity to $\theta >\xi$, and those not infected retain their level of immunity. An example function for *f* could be

5 $$\begin{aligned} f(\theta )= \left\{ \begin{array}{rcl} \dfrac{\left( \theta - \left( 1-\epsilon \right) x\right) ^{a}\left( \left( 1+\epsilon \right) x -\theta \right) ^{b}}{\left( 2\epsilon x\right) ^{a+b+1}B(a+1,b+1)}: &{} &{} \left( 1-\epsilon \right) x<\theta <\left( 1+\epsilon \right) x \\ 0: &{} &{} \text{ otherwise } \end{array} \right. \end{aligned}$$

for some $x<1/\left( 1+\epsilon \right)$. We have $\lim _{\epsilon \rightarrow 0}f(\theta )=\delta (x)$, and require $\epsilon <1$ to ensure $\theta >0$. An example function for *g* could be

6 $$\begin{aligned} g(\theta ,\xi )= \left\{ \begin{array}{rcl} \dfrac{\left( \theta - \left( 1-\epsilon \right) z(\xi )\right) ^{a}\left( \left( 1+\epsilon \right) z(\xi ) -\theta \right) ^{b}}{\left( 2\epsilon z(\xi )\right) ^{a+b+1}B(a+1,b+1)}: &{} &{} \left( 1-\epsilon \right) z(\xi )<\theta <\left( 1+\epsilon \right) z(\xi ) \\ 0: &{} &{} \text{ otherwise } \end{array} \right. \end{aligned}$$

where *z* is a function with $0<z(\xi )<1/\left( 1+\epsilon \right)$, and $z^{\prime }(\xi )>0$ to ensure that $\theta >\xi$. One suitable function would be $z(\xi )=x+\left( 1-x\right) \xi$, and more generally *x* could be a function of $\xi$. The function $h(\theta ,\xi )=\xi$. The actions of *f*, *g* and *h* are illustrated in Fig. 1b.

### The general case

In the general case an individual’s immunity level is increased at each infection until a maximum value $\theta =1$ is reached, those who were infected during the year increase their level of immunity to $\theta >\xi$, and those not infected reduce their level of immunity. The functions *f* and *g* could be as in the second example (Eqs. 5 and 6), and the function *h* could be as in the first example (Eq. 4). Their actions are illustrated in Fig. 1c.

## The deterministic continuous-discrete model of multi-season epidemics

For the situations considered in Sects. 2 and 3, if $\theta$ does not vary continuously at some initial time, $\theta$ can only take discrete values. Hence the model can be recast with *m* discrete compartments $S^\ell (t)$ instead of $S(t,\theta )$ depending on a continuous variable $\theta$. The function $k(\theta )$ is then replaced with a number of factors $k_\ell \leqslant 1$, with $k_{\ell +1}\leqslant k_\ell$. The functions $f(\theta )$, $g(\theta ,\xi )$, and $h(\theta ,\xi )$ that map points to PMFs are replaced with functions $F_\ell$, $G_\ell$ and $H_\ell$ that map points to points, as per the illustration in Fig. 1.

### Within-season

The within-season model becomes

$$\begin{aligned} \imath _\emptyset (t)&= \jmath _\emptyset (t) +\mathcal {R}_0S^\emptyset (t)\Lambda (t) \\ \imath _\ell (t)&=\jmath _\ell (t)+ \mathcal {R}_0k_\ell S^\ell (t) \Lambda (t) \qquad \ell =1\ldots m \\ \Lambda (t)&= \int _0^\infty p(t-\tau )\left( \imath _\emptyset (\tau )+\sum _{\ell =1}^m\imath _\ell (\tau )\right) \,\text{ d }\tau \end{aligned}$$

with

$$\begin{aligned} S^\emptyset (t)= S^\emptyset (0)-\int _0^t \imath _\emptyset (t^{\prime })\,\text{ d }t^{\prime } \qquad S^\ell (t) = S^\ell (0)-\int _0^t \imath _\ell (t^{\prime })\,\text{ d }t^{\prime } \end{aligned}$$

The epidemic *takes off* if $\mathcal {R}>1$ where

7 $$\begin{aligned} \mathcal {R}=\mathcal {R}_0\left( S^\emptyset (0)+\sum _{\ell =1}^m k_\ell S^\ell (0)\right) \end{aligned}$$

The total proportion of the population infected in the epidemic is

8 $$\begin{aligned} \mathcal {P}=S^\emptyset (0)-S^\emptyset (\infty )+\sum _{\ell =1}^m\left\{ S^\ell (0)-S^\ell (\infty )\right\} \end{aligned}$$

where $S^\emptyset (\infty )=S^\emptyset (0) e^{-\mathcal {R}_0\mathcal {P}}$, $S^\ell (\infty )=S^\ell (0) e^{-\mathcal {R}_0k_\ell \mathcal {P}}$, and the final size of the epidemic solves

9 $$\begin{aligned} \mathcal {P}&=S^\emptyset (0)\left( 1-e^{-\mathcal {R}_0\mathcal {P}}\right) +\sum _{\ell =1}^m S^\ell (0)\left( 1-e^{-\mathcal {R}_0k_\ell \mathcal {P}}\right) \nonumber \\&=1-e^{-\mathcal {R}_0\mathcal {P}}+\sum _{\ell =1}^m S^\ell (0)\left( e^{-\mathcal {R}_0\mathcal {P}}-e^{-\mathcal {R}_0k_\ell \mathcal {P}}\right) \end{aligned}$$

This is a generalisation of the model analysed in Roberts et al. (2019). Differentiating Eq. 9 by $S^j(0)$ for $j=1\ldots m$

$$\begin{aligned} \frac{\partial \mathcal {P}}{\partial S^j(0)} = \frac{e^{-\mathcal {R}_0\mathcal {P}}-e^{-\mathcal {R}_0k_j\mathcal {P}}}{1-\mathcal {R}_0e^{-\mathcal {R}_0\mathcal {P}}-\mathcal {R}_0\sum _{\ell =1}^m S^\ell (0)\left( k_\ell e^{-\mathcal {R}_0k_\ell \mathcal {P}}-e^{-\mathcal {R}_0\mathcal {P}}\right) } \end{aligned}$$

### Between-seasons

Let those who were infected for the first time this season enter the compartment $S^u$, those who were previously in compartment $S^\ell$ who were infected this season enter the compartment $S^{v(\ell )}$ with $v(\ell )\geqslant \ell$, and those who were previously in compartment $S^\ell$ who were not infected this season enter the compartment $S^{w(\ell )}$ with $w(\ell )\leqslant \ell$. In the absence of population turnover and antigenic drift, the proportion of the population in the $S^\sigma$ compartment at the beginning of the next season would be $F_\sigma +G_\sigma +H_\sigma$ where

$$\begin{aligned} F_\sigma = \left\{ \begin{array}{lcl} S^{\emptyset }(0)-S^{\emptyset }(\infty ) = S^{\emptyset }(0)\left( 1- e^{-\mathcal {R}_0\mathcal {P}}\right) : &{} &{} \sigma =u \\ \qquad \qquad \qquad \qquad \qquad \qquad \qquad \qquad \qquad \quad \, 0: &{} &{} \text{ otherwise } \end{array} \right. \end{aligned}$$

and

$$\begin{aligned} G_\sigma&= \sum _{\sigma =v(\ell )}\left( S^\ell (0)-S^\ell (\infty )\right) = \sum _{\sigma =v(\ell )}S^\ell (0)\left( 1-e^{-\mathcal {R}_0k_\ell \mathcal {P}}\right) \\ H_\sigma&= \sum _{\sigma =w(\ell )}S^\ell (\infty )= \sum _{\sigma =w(\ell )}S^\ell (0)e^{-\mathcal {R}_0k_\ell \mathcal {P}} \end{aligned}$$

To account for population turnover and antigenic drift, we allow a proportion $1-c$ of the population to be replaced with fully susceptible hosts between seasons. As in Sect. 3 we use the subscript *n* to signify this season, and $n+1$ for next season. The initial conditions for season $n+1$ are

$$\begin{aligned} S^\sigma _{n+1}(0) =c\left( F_\sigma +G_\sigma +H_\sigma \right) \qquad S^\emptyset _{n+1}(0) =1-\sum _{\sigma =1}^m S^\sigma _{n+1}(0) \end{aligned}$$

We can write this mapping in vector form. Define $\textbf{s}_n$ to be the vector whose $\ell ^{\text{ th }}$ component is $S_n^\ell (0)$ for $\ell =1\ldots m$, and $E(\textbf{s}_n)=e^{-\mathcal {R}_0\mathcal {P}_n}$ where $\mathcal {P}_n$ is the final size of the within season epidemic with initial conditions $\textbf{s}_n$. There is no need to explicitly model the initial condition for the fully susceptible host compartment, as $S^\emptyset _n(0)=1-\Vert \textbf{s}_n\Vert _1$. The mapping becomes

10 $$\begin{aligned} \textbf{s}_{n+1}=\textbf{C}(E(\textbf{s}_n))\textbf{s}_n+\textbf{q}(E(\textbf{s}_n)) \end{aligned}$$

where $\textbf{C}$ is an $m\times m$ matrix, and $\textbf{q}$ is an *m* dimensional vector valued function. The fixed point of the map solves

$$\begin{aligned} \textbf{s}^\star =\left( \textbf{I}-\textbf{C}(E^\star )\right) ^{-1}\textbf{q}(E^\star ) \end{aligned}$$

where $$E^\star =\exp (-\mathcal {R}_0\mathcal {P}^\star )$$ and

$$\begin{aligned} \mathcal {P}^\star =1-E^\star +\sum _{\ell =1}^m s_\ell ^\star \left( E^\star -E^{\star k_\ell }\right) \end{aligned}$$

Stability of the map depends on the eigenvalues of the Jacobian matrix, evaluated at $\textbf{s}=\textbf{s}^\star$. Differentiating Eq. 10 we find that $\textbf{J}$ has components

$$\begin{aligned} J_{\sigma j} (\textbf{s}_n,E(\textbf{s}_n))&=C_{\sigma j}+\sum _{\ell =1}^m \frac{\partial C_{\sigma \ell }}{\partial s_j}s_\ell +\frac{\partial q_\sigma }{\partial s_j} \\&=C_{\sigma j}-\mathcal {R}_0E\left( \sum _{\ell =1}^m \frac{\partial C_{\sigma \ell }}{\partial E}s_\ell +\frac{\partial q_\sigma }{\partial E}\right) \frac{\partial \mathcal {P}}{\partial s_j} \end{aligned}$$

where $s_\ell$ is the $\ell ^{\text{ th }}$ component of the vector $\textbf{s}_n$. The fixed point is stable if the eigenvalues of $\textbf{J}(\textbf{s}^\star ,E^\star )$ have absolute value less than one.

## Numerical simulations of the deterministic continuous-discrete model

We now explore four scenarios. For each we provide a general specification, with explicit expressions and numerical results for a host population that is either fully susceptible or has one of four levels of immunity ($m=4$). For the numerical results we present orbit diagrams showing the annual final size $\mathcal {P}$ (see Eq. 8) and effective reproduction number $\mathcal {R}$ (see Eq. 7) as the parameter $k_1$ varies from zero to one, with $k_2=k_1^2$, $k_3=k_1^3$ and $k_4=0$; with $c=0.9$ or $c=0.7$; and with $\mathcal {R}_0=2.0$ or $\mathcal {R}_0=4.0$. Recall that the parameter $k_\ell$ specifies the relative susceptibility of the compartment $S^\ell$ and *c* accounts for between season population turnover and antigenic drift. Hence $c=0.9$ implies that $10\%$ of the population is effectively replaced with fully susceptible hosts between each season, and $k_4=0$ means that the $S^4$ class is immune to infection. In most cases the initial conditions are $S_1^\emptyset (0)=1$, $S_1^\ell (0)=0$ for $\ell =1\ldots m$, but we also investigate alternative conditions to check for the existence of multiple attractors.

### The time since most recent infection determines susceptibility

For this example we choose $u=m$, $v(\ell )=m$ for all $\ell$ and $w(\ell )=\ell -1$ for $\ell =2\ldots m$. This leads to

$$\begin{aligned} S_{n+1}^\ell (0) =cS_n^{\ell +1}(0) e^{-\mathcal {R}_0k_{\ell +1}\mathcal {P}}: \quad \quad \ell =1,\ldots m-1 \end{aligned}$$

and

$$\begin{aligned} S_{n+1}^m(0)&=cS_n^\emptyset (0)\left( 1-e^{-\mathcal {R}_0\mathcal {P}}\right) +c \sum _{\ell =1}^{m-1}S_n^\ell (0) \left( 1-e^{-\mathcal {R}_0k_\ell \mathcal {P}} \right) \\&= c\left( 1-e^{-\mathcal {R}_0\mathcal {P}}\right) +c \sum _{\ell =1}^mS_n^\ell (0)\left( e^{-\mathcal {R}_0\mathcal {P}}-e^{-\mathcal {R}_0k_\ell \mathcal {P}} \right) \end{aligned}$$

where we have used $S_n^\emptyset (0)+\sum _{\ell =1}^{m}S_n^\ell (0) =1$ and $k_m=0$. The between season map $\textbf{s}_{n+1}=\textbf{C}(\textbf{s}_n)\textbf{s}_n+\textbf{q}(\textbf{s}_n)$ is defined as follows. The matrix $\textbf{C}(E)$ has components

$$\begin{aligned} C_{\sigma \ell }= \left\{ \begin{array}{rcl} cE^{k_\ell }: &{} &{} \sigma =\ell -1,\,\ell =2\ldots m \\ c\left( E-E^{k_\ell } \right) : &{} &{} \sigma =m,\,\ell =1\ldots m \\ 0: &{} &{} \text{ otherwise } \end{array} \right. \end{aligned}$$

and the vector $\textbf{q}(E)$ has all components zero apart from $q_m=c\left( 1-E\right)$. The fixed point has components

$$\begin{aligned} s_\sigma ^\star = \frac{s_1^\star }{c^{\sigma -1}\prod _{\ell =2}^\sigma E^{\star k_\ell }}:\quad \quad \sigma =2\ldots m \end{aligned}$$

and

$$\begin{aligned} s^\star _1=\dfrac{c\left( 1-E^\star \right) }{cE^{\star k_1}+\left( 1-cE^\star \right) \left( 1+\displaystyle \sum _{\sigma =2}^m\dfrac{1}{c^{\sigma -1}\prod _{\ell =2}^\sigma E^{\star k_\ell }}\right) } \end{aligned}$$

The map is illustrated for $m=4$ below (recall $E^{k_4}=1$).

$$\begin{aligned} \textbf{s}_{n+1}=c\left( \begin{array}{cccc}0 &{} E^{k_2} &{} 0 &{} 0 \\ 0 &{} 0 &{} E^{k_3} &{} 0 \\ 0 &{} 0 &{} 0 &{} 1 \\ E-E^{k_1} &{} E-E^{k_2} &{} E-E^{k_3} &{} E-1 \end{array}\right) \textbf{s}_n+\left( \begin{array}{c}0 \\ 0 \\ 0 \\ c\left( 1-E\right) \end{array}\right) \end{aligned}$$

The fixed point has components

$$\begin{aligned} s_2^\star = \frac{s_1^\star }{c E^{\star k_2}}\quad s_3^\star = \frac{s_1^\star }{c^2 E^{\star k_2}E^{\star k_3}}\quad s_4^\star = \frac{s_1^\star }{c^3 E^{\star k_2} E^{\star k_3}} \end{aligned}$$

**Fig. 2:**
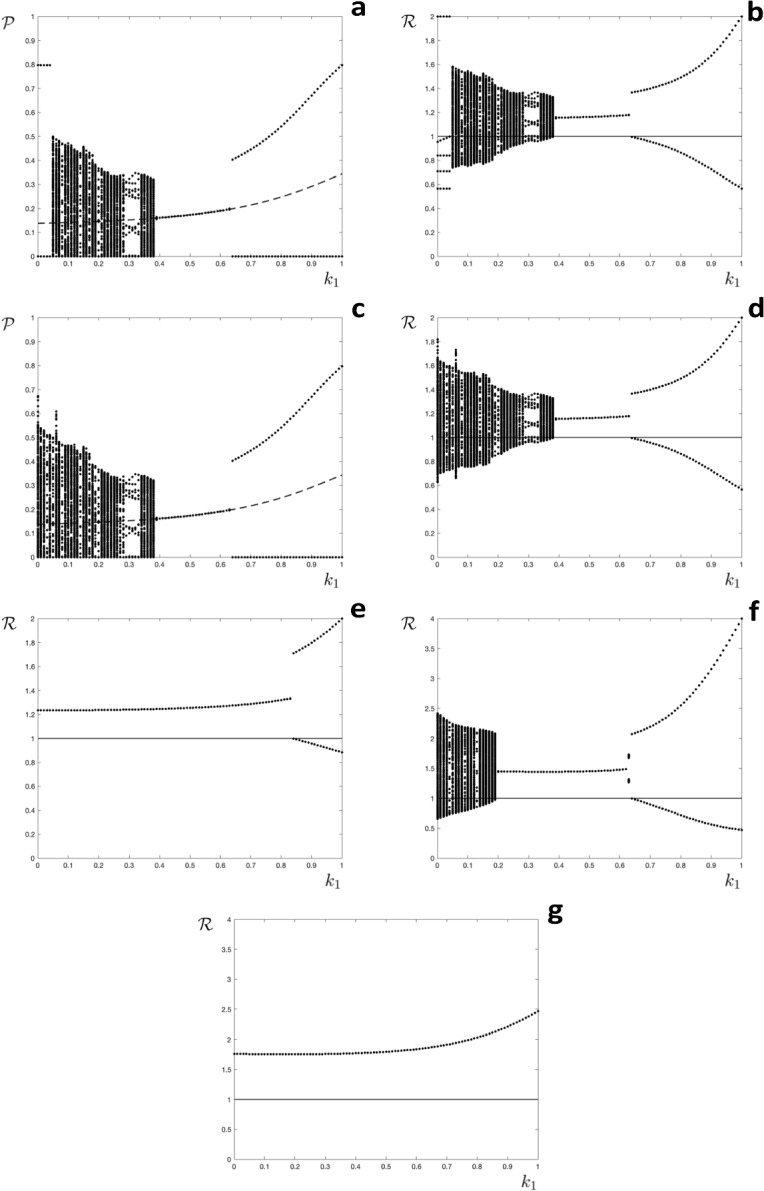
Orbit diagrams for the model where the time since last infection determines immunity. **a**, **c** the annual proportion of hosts infected $(\mathcal {P})$ as a function of the level of partial immunity $(k_1)$, the broken line shows an unstable fixed point; **b**, **d**–**g**: the effective reproduction number $(\mathcal {R})$ as a function of the level of partial immunity $(k_1)$, the horizontal line is at $\mathcal {R}=1$. **a**–**d**: $\mathcal {R}_0=2.0$, $c=0.9$. **e** $\mathcal {R}_0=2.0$, $c=0.7$. F: $\mathcal {R}_0=4.0$, $c=0.9$. **g** $\mathcal {R}_0=4.0$, $c=0.7$. Initial conditions **a**, **b**, **e**, **f**, **g**: $\textbf{s}_0=\textbf{0}=\left( 0,0,0,0\right) ^{\intercal }$, **c**, **d**: $\textbf{s}_0=\overline{\textbf{s}}=\left( 0.3,0.2,0.1,0\right) ^{\intercal }$

and

$$\begin{aligned} s^\star _1=\dfrac{c\left( 1-E^\star \right) }{cE^{*k_1}+\left( 1-cE^\star \right) \left( 1+ \dfrac{1}{cE^{\star k_2} } + \dfrac{1}{c^2 E^{\star k_2} E^{\star k_3}}+ \dfrac{1}{c^3 E^{\star k_2} E^{\star k_3}}\right) } \end{aligned}$$

Numerical results are presented in Fig. 2. The orbit diagram Fig. 2a shows the proportion of the population infected each year $\mathcal {P}$ as $k_1$ varies from zero to one. The diagram reveals that solutions tend to a fixed point (same size epidemic each year) for $0.39<k_1<0.63$, but have complicated (high-period, non-recurrent or chaotic) dynamics for $0.06\leqslant k_1\leqslant 0.39$. For $k_1>0.63$ if an epidemic occurs it has the same size as in previous years, but some years there is no epidemic. Figure 2b shows the variability of the effective reproduction number $\mathcal {R}$ as $k_1$ varies from zero to one. The figure reveals that for $k_1>0.63$ there is an epidemic in alternate years, but for $k_1<0.06$ there is a five year cycle, with four years without an epidemic, followed by an epidemic in the fifth year. Figure 2c, d show the dynamics with the same parameter values but with a different set of initial conditions. These diagrams reveal the existence of an alternative attractor for small values of $k_1$. Figure 2e–g show the dynamics with alternative values of $\mathcal {R}_0$ or *c*. For these figures only the dynamics of the effective reproduction number $\mathcal {R}$ are shown. No sensitivity on initial conditions was observed with these parameter values. A region of complicated dynamics was observed for the example with $c=0.9$ and $\mathcal {R}_0=4.0$ (Fig. 2f), but no such region was found for the examples with $c=0.7$ (Fig. 2e, g).

### The number of infections determines susceptibility

We define a finite number of compartments $S^\ell$, with $1\leqslant \ell \leqslant m$. If each infection results in a host increasing its immunity to the next level, and not being infected results in immunity staying at the same level, then we have $u=1$, $v(\ell )=\ell +1$ for $\ell =1\ldots m-1$ and $w(\ell )=\ell$ for $\ell =1\ldots m$. This leads to

$$\begin{aligned} S_{n+1}^\ell (0) =c\left( S_n^{\ell -1}(0) \left( 1-e^{-\mathcal {R}_0k_{\ell -1} \mathcal {P}} \right) + S_n^{\ell }(0) e^{-\mathcal {R}_0k_{\ell }\mathcal {P}} \right) \quad : \quad \ell =2,\ldots m-1 \end{aligned}$$

and

$$\begin{aligned} S_{n+1}^1(0)&=c\left( \left( 1-\sum _{\ell =1}^{m}S_n^\ell (0) \right) \left( 1-e^{-\mathcal {R}_0\mathcal {P}} \right) + S_n^1(0) e^{-\mathcal {R}_0k_{1}\mathcal {P}} \right) \\ S_{n+1}^m(0)&=cS_n^{m-1}(0) \left( 1-e^{-\mathcal {R}_0k_{m-1} \mathcal {P}} \right) +c \end{aligned}$$

where we have used $S_n^\emptyset (0)+\sum _{\ell =1}^{m}S_n^\ell (0) =1$ and $k_m=0$. The between season map $\textbf{s}_{n+1}=\textbf{C}(\textbf{s}_n)\textbf{s}_n+\textbf{q}(\textbf{s}_n)$ is defined as follows. The matrix $\textbf{C}(E)$ has components

$$\begin{aligned} C_{\sigma \ell }= \left\{ \begin{array}{lcl} c\left( E-1+E^{k_1}\right) &{}: &{} \sigma =\ell =1 \\ c\left( E-1\right) &{}: &{} \sigma =1,\,\ell =2\ldots m \\ cE^{k_\ell } &{}: &{} \sigma =\ell ,\,\ell =2\ldots m \\ c\left( 1-E^{k_\ell } \right) &{}: &{} \sigma =\ell +1,\,\ell =1\ldots m-1 \\ 0 &{}: &{} \text{ otherwise } \end{array} \right. \end{aligned}$$

and the vector $\textbf{q}(E)$ has all components zero apart from $q_1=c\left( 1-E\right)$. The map is illustrated for $m=4$ below.

$$\begin{aligned} \textbf{s}_{n+1}=c\left( \begin{array}{cccc}E-1+E^{k_1} &{} E-1 &{} E-1 &{} E-1 \\ 1-E^{k_1} &{} E^{k_2} &{} 0 &{} 0 \\ 0 &{} 1-E^{k_2} &{} E^{k_3} &{} 0 \\ 0 &{} 0 &{} 1-E^{k_3} &{} 1 \end{array}\right) \textbf{s}_n+\left( \begin{array}{c}c\left( 1-E\right) \\ 0 \\ 0 \\ 0\end{array}\right) \end{aligned}$$

A fixed point of this map is

$$\begin{aligned} s^\star _2&=f_2(E^\star )s^\star _1=\frac{c\left( 1-E^{\star k_1}\right) }{1-cE^{\star k_2}}s^\star _1 \\ s^\star _3&=f_3(E^\star )s^\star _1 =\frac{c^2\left( 1-E^{\star k_1}\right) \left( 1-E^{\star k_2}\right) }{\left( 1-cE^{\star k_2}\right) \left( 1-cE^{\star k_3}\right) }s^\star _1 \\ s^\star _4&=f_4(E^\star )s^\star _1 =\frac{c^3\left( 1-E^{\star k_1}\right) \left( 1-E^{\star k_2}\right) \left( 1-E^{\star k_3}\right) }{\left( 1-cE^{\star k_2}\right) \left( 1-cE^{\star k_3}\right) \left( 1-c\right) }s^\star _1 \end{aligned}$$

where $s^\star _1$ can be found by substituting in

$$\begin{aligned} s^\star _1=c\left( 1-E^\star \right) \left( 1-s_1^\star -s_2^\star -s_3^\star -s_4^\star \right) +cE^{\star k_1}s_1^\star \end{aligned}$$

to obtain

$$\begin{aligned} s^\star _1=\frac{c\left( 1-E^\star \right) }{1-cE^{\star k_1}+c\left( 1-E^\star \right) \left( 1+f_2(E^\star )+f_3(E^\star )+f_4(E^\star )\right) } \end{aligned}$$

**Fig. 3:**
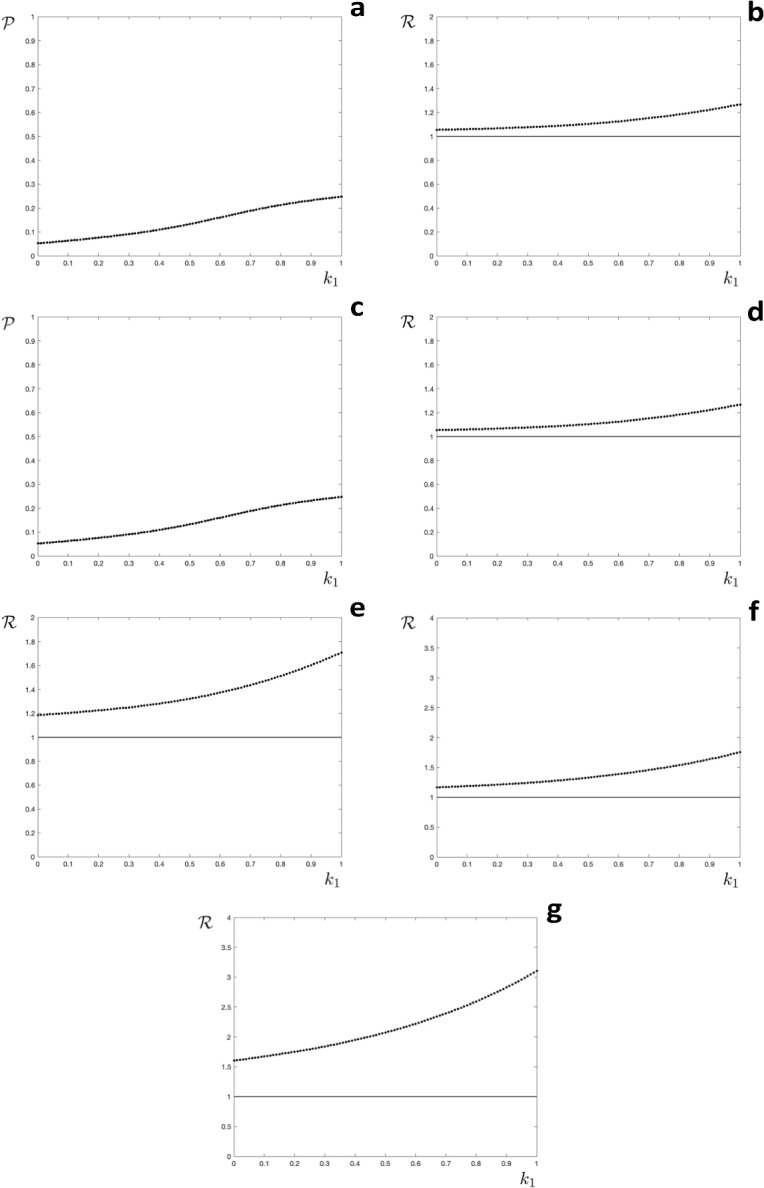
Orbit diagrams for the model where the number of infections determines immunity. **a**, **c** the annual proportion of hosts infected $(\mathcal {P})$ as a function of the level of partial immunity $(k_1)$, the broken line shows an unstable fixed point; **b**, **d**–**g**: the effective reproduction number $(\mathcal {R})$ as a function of the level of partial immunity $(k_1)$, the horizontal line is at $\mathcal {R}=1$. **a**–**d** $\mathcal {R}_0=2.0$, $c=0.9$. **e**: $\mathcal {R}_0=2.0$, $c=0.7$. **f** $\mathcal {R}_0=4.0$, $c=0.9$. **g**: $\mathcal {R}_0=4.0$, $c=0.7$. Initial conditions **a**, **b**, **e**–**g**: $\textbf{s}_0=\left( 0,0,0,0\right) ^{\intercal }$, C,D: $\textbf{s}_0=\left( 0.3,0.2,0.1,0\right) ^{\intercal }$

Numerical results are presented in Fig. 3. The figure is structured to enable direct comparison with the results shown in Fig. 2. In particular, for all of the parameter values chosen the attractor was a fixed point (period one) and no dependency on initial conditions was observed.

**Fig. 4:**
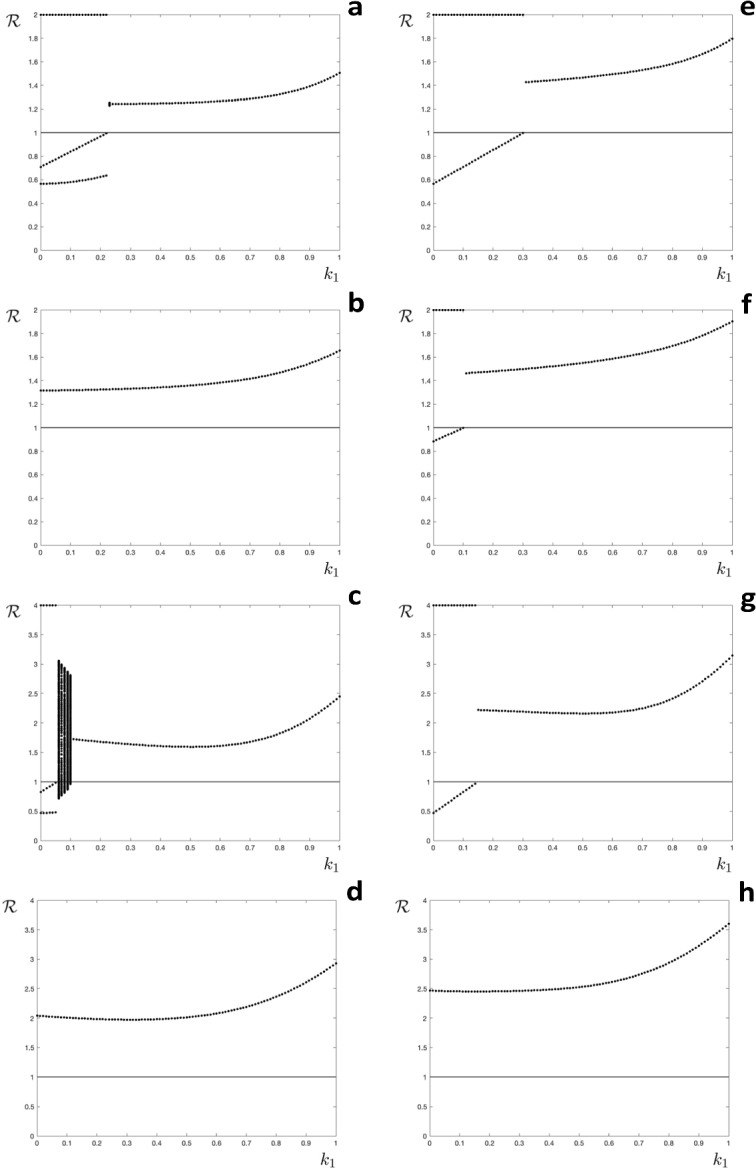
Orbit diagrams for the general model. The effective reproduction number $(\mathcal {R})$ as a function of the level of partial immunity $(k_1)$. **a**–**d** Having been infected increases immunity more than the loss due to escaping infection. **e**–**h** Having been infected increases immunity less than the loss due to escaping infection. **a**, **e** $\mathcal {R}_0=2.0$, $c=0.9$. **b**, **f** $\mathcal {R}_0=2.0$, $c=0.7$. **c**, **g** $\mathcal {R}_0=4.0$, $c=0.9$. **d**, **h**: $\mathcal {R}_0=4.0$, $c=0.7$. Initial conditions $\textbf{s}_0=\left( 0,0,0,0\right) ^{\intercal }$, the horizontal line is at $\mathcal {R}=1$

### The number of infections and the time since last infection determine susceptibility

We define a finite number of compartments $S^\ell$, with $1\leqslant \ell \leqslant m$. Then define $u>0$, $v(\ell )\geqslant \ell$ with $v(m)=m$, and $w(\ell )< \ell$; *u*, *v*, *w* all integer.

First we consider a scenario where infection increases immunity more than non-infection results in a decrease. For example, suppose that having been infected increases the immunity level by two steps, but without infection immunity drops one level between seasons. We would then have $u=2$, $v(\ell )=\ell +2$ for $\ell =1\ldots m-2$, $v(m-1)=m$, and $w(\ell )=\ell -1$ for $\ell >1$. With $m=4$.

$$\begin{aligned} \textbf{s}_{n+1}=c\left( \begin{array}{cccc}0 &{} E^{k_2} &{} 0 &{} 0 \\ E-1 &{} E-1 &{} E-1+E^{k_3} &{} E-1 \\ 1-E^{k_1} &{} 0 &{} 0 &{} E^{k_4} \\ 0 &{} 1-E^{k_2} &{} 1-E^{k_3} &{} 0 \end{array}\right) \textbf{s}_n+\left( \begin{array}{c} 0 \\ c\left( 1-E\right) \\ 0 \\ 0\end{array}\right) \end{aligned}$$

Numerical results are presented in Fig. 4a–d. We only present orbit diagrams for the changes in effective reproduction number $\mathcal {R}$, as these are sufficient to provide a description of the dynamics. It can be seen that for the examples with $c=0.9$ (Fig. 4a, c) there is a period three solution for low values of $k_1$, comprising two seasons with no epidemic ($\mathcal {R}<1$) followed by one season with an epidemic. For the example with $\mathcal {R}_0=4.0$ and $c=0.9$ there is also a region of $k_1$ with complicated (high period) dynamics (Fig. 4c). For all other examples and values of $k_1$ the observed attractor is a period one solution (fixed point of the discrete map).

Now consider a scenario where infection increases immunity less than non-infection results in a decrease. For example having been infected increases the immunity level by one step, but without infection immunity drops two levels between seasons. We would then have $u=1$, $v(\ell )=\ell +1$ for $\ell =1\ldots m-1$, and $w(\ell )=\ell -2$ for $\ell >2$. For example, with $m=4$.

$$\begin{aligned} \textbf{s}_{n+1}=c\left( \begin{array}{cccc}E-1 &{}\quad E-1 &{}\quad E-1+E^{k_3} &{}\quad E-1 \\ 1-E^{k_1} &{}\quad 0 &{}\quad 0 &{}\quad E^{k_4} \\ 0 &{}\quad 1-E^{k_2} &{}\quad 0 &{}\quad 0 \\ 0 &{}\quad 0 &{}\quad 1-E^{k_3} &{}\quad 0 \end{array}\right) \textbf{s}_n+\left( \begin{array}{c} c\left( 1-E\right) \\ 0 \\ 0 \\ 0\end{array}\right) \end{aligned}$$

Numerical results are presented in Fig. 4e–h. The orbit diagrams show epidemics in alternate years for some values of $k_1$, otherwise the fixed point is stable with an epidemic every year. None of the orbit diagrams show complicated high period dynamics.

### Adding a stochastic component

**Fig. 5:**
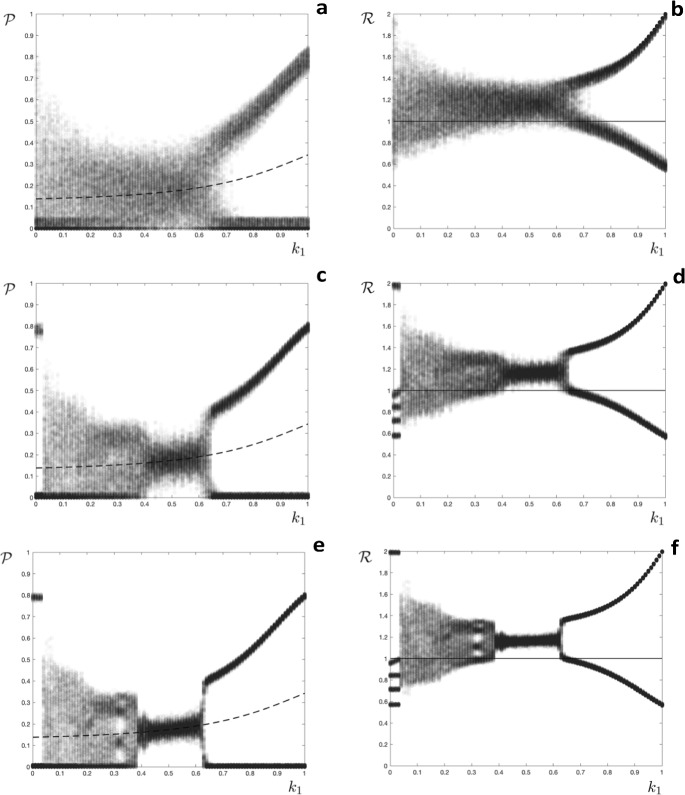
Orbit diagrams for the model where the time since last infection determines immunity and stochastic perturbations have been added. A,C,E: the annual proportion of hosts infected $(\mathcal {P})$ as a function of the level of partial immunity $(k_1)$, the broken line shows an unstable fixed point of the corresponding deterministic model; B,D,F: the effective reproduction number $(\mathcal {R})$ as a function of the level of partial immunity $(k_1)$, the horizontal line is at $\mathcal {R}=1$. A,B: $\delta =0.05$; C,D: $\delta =0.02$; E,F: $\delta =0.01$. Initial conditions $\textbf{s}_0=\left( 0,0,0,0\right) ^{\intercal }$

In order to approximate solutions of the stochastic model, we simulated a hybrid model by calculating the final size of the deterministic within-season model ($\mathcal {P}$, Eq. 9) and then adding a small perturbation $\mathcal {P}\rightarrow \mathcal {P}+\delta \mathcal {P}$. The magnitude of the perturbation was taken at random from a uniform distribution $\delta \mathcal {P}\in \left[ -\delta ,\delta \right]$, truncated if necessary so that $\mathcal {P}\in \left[ 0,1\right]$. Realisations of the orbit diagrams for the model with immunity determined by the time since last infection, as presented in Fig. 2a and b, but now with the perturbations $\delta \mathcal {P}$ added at each season, are presented in Fig. 5. Comparing Fig. 2a, b with Fig. 5 we see that when $\delta =0.05$ the stochastic perturbation obscures the period five solution for $k_1<0.06$ and the period one solution for $0.39<k_1<0.63$ (Fig. 5a, b). Orbits in these regions become virtually indistinguishable from those in the region of $k_1$ that leads to complicated dynamics. The period five and period one solutions are discernible when $\delta =0.02$ (Fig. 5c, d) and are clearly visible when $\delta =0.01$ (Fig. 5e, f). In all of the orbit diagrams shown in Fig. 5 the period two solutions for $k_1>0.63$ are preserved.

## Discussion

We previously investigated a model for a seasonal infectious disease where having been infected in one season an individual host was fully protected for the next season, and partially protected for the season after, before becoming fully susceptible again if not infected (Roberts et al. 2019). That model exhibited complicated dynamics for a range of parameter values, as have a number of other models for seasonal infections with different structures (Andreasen 2003; Bacaer and Ouifki 2007; Earn et al. 2000; He and Earn 2007; Mathews et al. 2007; Viboud et al. 2006). The focus of our present study was to investigate if different dynamics would be observed if the level of immunity was determined by the elapsed time since a host was last infected, or by the number of infections a host had experienced. When immunity was determined by the time since infection we observed complicated dynamics over a range of parameter values, especially if acquired immunity resulted in stronger protection (lower values of $k_1$ in Fig. 2). In contrast, when immunity was determined by the number of infections experienced the model exhibited a steady state, corresponding to an epidemic of the same size every year (Fig. 3). When the two mechanisms were combined the steady state attractor was predominant, although periodic or complicated dynamics were observed for lower values of $k_1$ in some instances (Fig. 4). These findings have potential implications for predictive modelling studies. For example, our results suggest that if the level of host immunity depends on the elapsed time since the last infection then the epidemiological dynamics may be unpredictable.

The model as presented includes the assumption that epidemiological parameters, and hence $\mathcal {R}_0$ are constant from season to season. However, virus evolution may result in variants presenting with different transmissibility in subsequent seasons. Similarly, the factor allowing for population turnover and antigenic drift has been taken as constant, but for influenza it has been shown that drift can be punctuated by some years of increased change (Smith et al. 2004). Should Covid-19 become seasonal then it is likely that a similar phenomenon would be observed, as different variants of the virus have already exhibited different transmissibility (Manathunga et al. 2023).

Where annual epidemics involve multiple strains of a virus, the timing of the introduction of each strain influences the within season dynamics (Roberts 2012). This would be another source of unpredictability when modelling the long term dynamics of seasonal infections such as influenza, for which some degree of cross-protection between strains has been determined (Ferguson et al. 2003). Modelling epidemics with multiple interacting viruses introduces complications (Gog and Grenfell 2002) and is the subject of ongoing research.

## Appendix A: The final size of the within-season model

Define the Laplace transform of a function $y(t,\theta )$ by

$$\begin{aligned} \overline{y}(s,\theta )=\mathcal {L}\left\{ y(t,\theta )\right\} =\int _0^\infty e^{-s t}y(t,\theta )\,\text{ d }t \end{aligned}$$

Taking transforms of Eqs. 1 and 2

$$\begin{aligned} \mathcal {L}\left\{ \frac{ \imath _\emptyset (t) }{S^\emptyset (t)}\right\}&=\mathcal {L}\left\{ \frac{ \jmath _\emptyset (t) }{S^\emptyset (t)}\right\} + \mathcal {R}_0\overline{\Lambda }(s) \\ \mathcal {L}\left\{ \frac{\imath (t,\theta )}{S(t,\theta )}\right\}&=\mathcal {L}\left\{ \frac{\jmath (t,\theta )}{S(t,\theta )}\right\} + \mathcal {R}_0k(\theta ) \overline{\Lambda }(s) \\ \overline{\Lambda }(s)&=\overline{p}(s)\left( \overline{\imath _\emptyset }(s) +\int _0^1\overline{\imath }(s,\theta )\,\text{ d }\theta \right) \end{aligned}$$

Substituting for $\imath _\emptyset (t)$ and $\imath (t,\theta )$ on the left hand side only

$$\begin{aligned} \mathcal {L}\left\{ -\frac{ 1 }{S^\emptyset (t)}\frac{\partial S^\emptyset }{\partial t}\right\}&=\mathcal {L}\left\{ \frac{ \jmath _\emptyset (t) }{S^\emptyset (t)}\right\} + \mathcal {R}_0\overline{\Lambda }(s) \\ \mathcal {L}\left\{ -\frac{1}{S(t,\theta )}\frac{\partial S}{\partial t}\right\}&=\mathcal {L}\left\{ \frac{\jmath (t,\theta )}{S(t,\theta )}\right\} + \mathcal {R}_0k(\theta ) \overline{\Lambda }(s) \end{aligned}$$

Now let $s\rightarrow 0$. We have $\overline{p}(0)=\int _0^\infty p(t)\,\text{ d }t=1$,

$$\begin{aligned} \lim _{s\rightarrow 0} \mathcal {L}\left\{ -\frac{1}{S^\emptyset (t)}\frac{\partial S^\emptyset }{\partial t}\right\}&=- \int _0^\infty \frac{1}{S^\emptyset (t)}\frac{\partial S^\emptyset }{\partial t}\,\text{ d }t= \log \left( \frac{S^\emptyset (0)}{S^\emptyset (\infty )}\right) \\ \lim _{s\rightarrow 0} \mathcal {L}\left\{ -\frac{1}{S(t,\theta )}\frac{\partial S}{\partial t}\right\}&=- \int _0^\infty \frac{1}{S(t,\theta )}\frac{\partial S}{\partial t}\,\text{ d }t= \log \left( \frac{S(0,\theta )}{S(\infty ,\theta )}\right) \\ \lim _{s\rightarrow 0}\overline{\imath _\emptyset }(s)&=\int _0^\infty \imath _\emptyset (t)\,\text{ d }t= S^\emptyset (0)-S^\emptyset (\infty ) \\ \lim _{s\rightarrow 0}\overline{\imath }(s,\theta )&=\int _0^\infty \imath (t,\theta )\,\text{ d }t= S(0,\theta )-S(\infty ,\theta ) \\ \lim _{s\rightarrow 0} \overline{\Lambda }(s)&=\int _0^\infty \Lambda (t) \,\text{ d }t \end{aligned}$$

The total proportion of the population infected in the epidemic is

$$\begin{aligned} \mathcal {P}=\int _0^\infty \Lambda (t) \,\text{ d }t =S^\emptyset (0)-S^\emptyset (\infty )+\int _0^1\left\{ S(0,\theta )-S(\infty ,\theta )\right\} \,\text{ d }\theta \end{aligned}$$

Consider now the imported cases, as $\dfrac{\jmath _\emptyset (t)}{S^\emptyset (t)}$ and $\dfrac{\jmath (t,\theta )}{S(t,\theta )}$ are assumed to be small, we neglect the quantities

$$\begin{aligned} \lim _{s\rightarrow 0}\mathcal {L}\left\{ \dfrac{\jmath _\emptyset (t)}{S^\emptyset (t)}\right\} =\int _0^\infty \dfrac{\jmath _\emptyset (t)}{S^\emptyset (t)}\,\text{ d }t \qquad \lim _{s\rightarrow 0}\mathcal {L}\left\{ \frac{\jmath (t,\theta )}{S(t,\theta )}\right\} =\int _0^\infty \frac{\jmath (t,\theta )}{S(t,\theta )}\,\text{ d }t \end{aligned}$$

We now have $S^\emptyset (\infty )=S^\emptyset (0) e^{-\mathcal {R}_0\mathcal {P}}$ and $S(\infty ,\theta )=S(0,\theta ) e^{-\mathcal {R}_0k(\theta )\mathcal {P}}$, and the final size of the epidemic solves Eq. 3.
